# Ortner’s syndrome secondary to thoracic aortic aneurysm: a case series

**DOI:** 10.1186/s13019-022-02023-1

**Published:** 2022-10-20

**Authors:** Zhiwen Zhang, Hai Feng, Xueming Chen, Wenrui Li

**Affiliations:** Department of Vascular Surgery, Beijing Friendship Hospital, Capital Medical University, Beijing, 100050 China

**Keywords:** Thoracic aortic aneurysm, Ortner’s syndrome, Thoracic endovascular aortic repair

## Abstract

**Background:**

Ortner’s syndrome refers to vocal cord paralysis resulting from compression of the left recurrent laryngeal nerve by abnormal mediastinal vascular structures. This retrospective case series details our experience with Ortner’s syndrome due to thoracic aortic aneurysm.

**Methods:**

This study was a retrospective analysis of a case series. A total of 4 patients (mean age, 65.5 years) with Ortner’s syndrome due to thoracic aortic aneurysm underwent thoracic endovascular aortic repair from July 2014 to May 2020. The patients’ demographics, comorbidities, initial symptoms, time from hoarseness to treatment, aneurysm shape and size, surgical procedures and outcome are summarized.

**Results:**

A total of 4 patients with Ortner’s syndrome due to thoracic aortic aneurysm were analyzed. All the patients underwent thoracic endovascular aortic repair with no complications during the hospitalization period. At a mean follow-up of 26.8 (8–77) months, hoarseness in 3 patients had completely resolved or improved, and the symptoms in 1 patient had not progressed.

**Conclusions:**

Hoarseness due to left recurrent laryngeal nerve palsy can be the presenting symptom of thoracic aortic aneurysm. Early diagnosis leads to timely treatment of these patients which may be helpful in the functional recovery of symptoms.

## Background

Hoarseness is a common clinical symptom that frequently has a benign cause. However, persistent or sudden hoarseness without a clear cause may indicate serious pathology and further investigation is required. Ortner’s syndrome is a rare cause of hoarseness, which refers to vocal cord paralysis caused by compression of the left recurrent laryngeal nerve (LRLN) due to abnormalities in the cardiovascular structure in the mediastinum [1]. Here, we present 4 cases of hoarseness associated with thoracic aortic aneurysms (TAA), which caused paralysis of the LRLN.

This report describes our experience in the diagnosis and treatment of Ortner’s syndrome caused by TAA. The patient’s characteristics, symptoms, treatment and outcome are presented and discussed.

## Methods

This is a case series of patients who had vocal cord paralysis caused by compression of the LRLN due to TAA. Our inclusion criteria were TAA patients of both genders who presented with hoarseness. Exclusion criteria included patients who had other causes of hoarseness such as neoplasia, iatrogenic procedures and other cardiovascular pathology. The primary outcome was recovery of hoarseness. The secondary outcomes included complications and mortality during the hospitalization period. Hoarseness was defined as changes in voice quality, pitch and loudness. All patients agreed to the intervention and underwent thoracic endovascular aortic repair (TEVAR) combining a chimney graft and hybrid repair of the TAA. Planning was performed based on prior assessment of patients’ general condition, their preferences and a signed written consent.

## Results

A series of 4 patients with Ortner’s syndrome due to TAA in our department from July 2014 to May 2020 were included in this study. Of these 4 patients, 2 were men, ranging in age from 54 to 76 years. The aneurysm was mainly from the arch and proximal descending aorta. Table 1 summarizes the patients’ demographics, comorbidities, initial symptoms, time from hoarseness to treatment, aneurysm shape and size, surgical procedures and outcome.

**Table 1:** Summary of the patients’ demographics, comorbidities, initial symptoms, time from hoarseness to treatment, aneurysm shape and size, surgical procedures and outcome

Case No) Gender /Age (yrs)	Comorbidities	Symptoms	Time from hoarseness to treatment (months)	Type of thoracic aneurysm/size (cm)	Proximal landing zone/type of debranching	Outcome
(1) Male/68	COPD, GERD, smoking	Hoarseness	1	Saccular/5/3.9	TEVAR/ zone 3/None	Completely resolved
(2) Male/64	COPD, smoking	Hoarseness	24	Saccular/6	TEVAR/zone 1/chimney graft of LCCA & LSA	No improvement of hoarseness
(3) Female/76	HT	Hoarseness	9	Saccular/5.4	TEVAR/ zone 2/None	Improvement of hoarseness
(4) Female/54	HT, CAD, DM, Pemphigus, Oral prednisone	Hoarseness	8	Fusiform/9.7	TEVAR/zone 1/carotid-carotid & carotid- LSA bypass	Improvement of hoarseness

COPD, Chronic obstructive pulmonary disease; GERD, Gastroesophageal reflux disease; HT, Hypertension; CAD, Coronary artery disease; DM, Diabetes mellitus

**Fig. 1:**
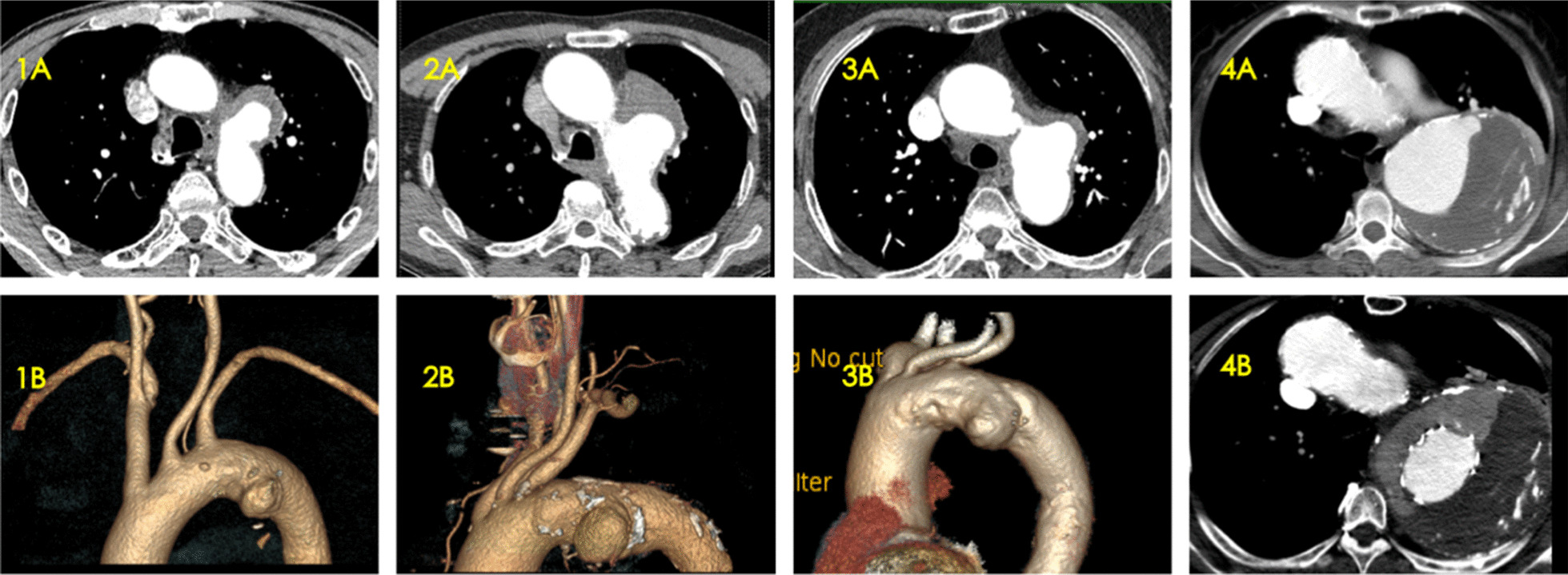
Computed tomography findings. **1A**, **2A**, **3A**: Axial view computed tomography scans of case 1–3; **1B, 2B, 3B**: 3D rendering of computed tomography scans of case 1–3; **4A**: Axial view computed tomography scans of case 4 before TEVAR (without symptoms of hoarseness); **4B**: Axial view computed tomography scans of case 4, 7 months after TEVAR (with symptoms of hoarseness), with type Ia endoleak and increased diameter of the aneurysm. TEVAR, Thoracic endovascular aortic repair

The initial symptom in most patients was hoarseness (3/4), ranging from altered voice quality, pitch and loudness. These 3 patients all attended the Otolaryngology Department first. The laryngeal examination revealed a left vocal cord paralysis in the paramedian position. An aorta-related examination was not ordered as the patients had no other symptoms such as chest pain or dysphagia. One month, 9 months, and 24 months after hoarseness appeared, respectively, these 3 patients were diagnosed with TAA by computed tomography scans and stent-graft repair of their thoracic aneurysms was performed (Fig. 1). The remaining patient was diagnosed with TAA during a routine health examination and was without symptoms of hoarseness. She was treated with conventional TEAVR. Hoarseness occurred 1 month after the operation, but the cause was not further clarified. Seven months later, she attended as an outpatient with severe hoarseness, and further investigation identified a type Ia endoleak, and the diameter of the aneurysm had increased significantly (Fig. 1). Hybrid aortic arch debranching (carotid-carotid bypass combined with left carotid-axillary bypass) and TEVAR were performed.

At a mean follow-up of 26.8 (8–77) months, hoarseness in 1 patient had completely resolved within 6 months after surgery, and hoarseness had improved in 2 patients at 3 months and 6 months, respectively, but was not fully resolved. Symptoms had not progressed in the remaining patient.

## Discussion

Ortner’s syndrome is a rare cause of left recurrent laryngeal nerve palsy. The enlargement of any intrathoracic structure close to the recurrent laryngeal nerve such as left atrial enlargement, dilatation of the left pulmonary artery, or TAA can result in compressive nerve palsy [2, 3]. In the superior mediastinum, the LRLN is a branch of the vagus nerve that descends antero-lateral to the aortic arch, passes deep to the ligamentum arteriosum and then ascends in the tracheo-esophageal groove. The nerve can be compressed by a widening aortic aneurysm sac at the aorta [4]. Three of our patients had hoarseness as the first symptom, and their aneurysms had mainly arisen from the arch and proximal descending aorta. In the remaining patient, the position where the diameter of aneurysm increased due to endoleak was similar. This may be related to the anatomical position of the recurrent laryngeal nerve.

Generally, patients with hoarseness visit the otolaryngologist for evaluation. Although, laryngoscopy can easily diagnose vocal cord paralysis, the differential diagnosis can be challenging [5]. Most vocal cord paralysis is due to neoplasia, followed by iatrogenic procedures, and intracranial diseases or aortic diseases also need to be considered. In aortic disease, in addition to TAA, mycotic aneurysm and aortic dissection may also cause hoarseness, and the latter two disorders have other symptoms [2, 4, 6]. Most TAAs are asymptomatic, but some TAA patients may have other symptoms including dysphagia due to compression of the esophagus and breathing difficulties due to incomplete opening of the glottis [7]. Therefore, when a common cause of hoarseness is not found, beware of hoarseness caused by compression of the LRLN, and a chest CT or X-ray may be ordered.

The management of aortic aneurysm disease mainly includes stent-graft repair of thoracic aneurysms and open surgery with aorta replacement [8]. According to the patient's condition, our patients opted for TEVAR, combining a chimney graft and hybrid repair of the TAA. Early diagnosis of Ortner’s syndrome may be helpful in starting immediate treatment to restore vocal cord function and prevent permanent damage to the LRLN. Voice improvement is expected within a few weeks of surgery and hoarseness has been reported to resolve completely within 4 months after surgery [7]. Endovascular stent grafting of the underlying TAA causes progressive shrinkage of the excluded and thrombosed aneurysm reducing nerve compression leading to resolution of the LRLN palsy.

In our patients, 1 patient’s hoarseness fully recovered, 2 patients had partial improvement of hoarseness, and hoarseness was still present in 1 patient. The patients who were treated earlier recovered better (Table 1). The patient with no improvement received treatment 2 years later after hoarseness onset. These results indicate that for patients with Ortner’s syndrome secondary to TAA, early treatment may be beneficial in restoring vocal cord function.

## Conclusion

We advocate vigilance in patients with hoarseness and the possibility of Ortner’s syndrome secondary to TAA should be considered and evaluated. For patients with TAA or treated with TEVAR surgery, a sudden onset of vocal cord disorder can suggest aneurysm diameter enlargement or endoleak. Early diagnosis leads to timely treatment of these patients which may be helpful in functional recovery of the LRLN.

## Data Availability

The datasets used and analyzed during the current study are available from the corresponding author on reasonable request.

## Abbreviations

LRLN: Left recurrent laryngeal nerve
TAA: Thoracic aortic aneurysms
TEVAR: Thoracic endovascular aortic repair
